# Supplementary material for: Screening and verification of proteins that interact with the anthocyanin-related transcription factor PbrMYB114 in ‘Yuluxiang’ pear
Source: PeerJ. 2024 Jun 14;12:e17540. doi: 10.7717/peerj.17540 (PMC11182023; doi:10.7717/peerj.17540)
Supplement: Supplemental Information 1 [file peerj-12-17540-s001.docx]

**Figure S1. Confirmation of positive interactors**


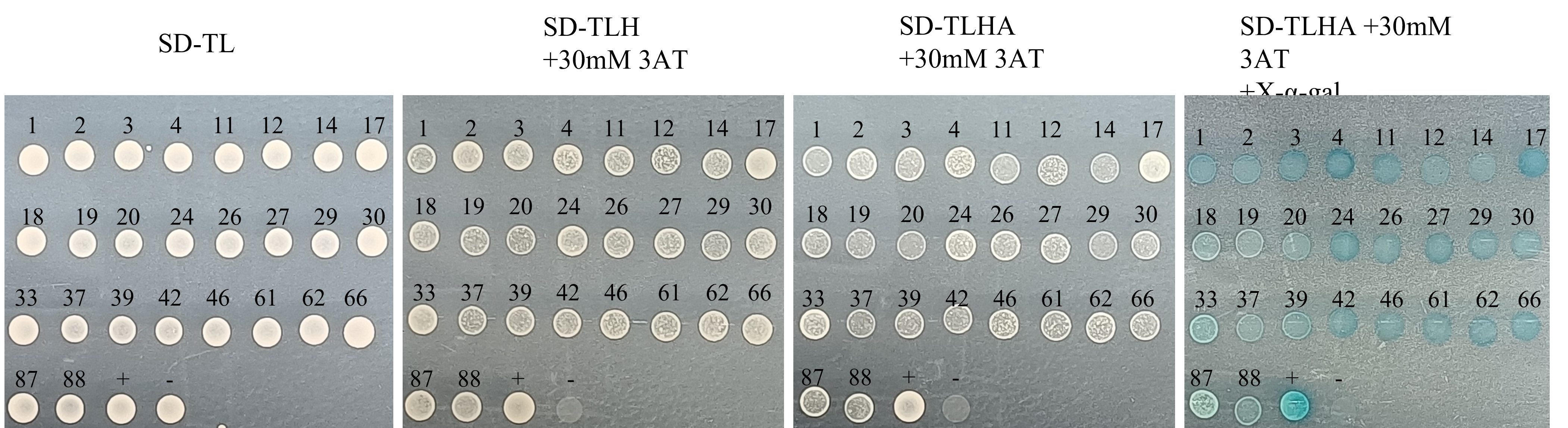


**Figure S2. PbrMYB114 interacts physically with a number of proteins**


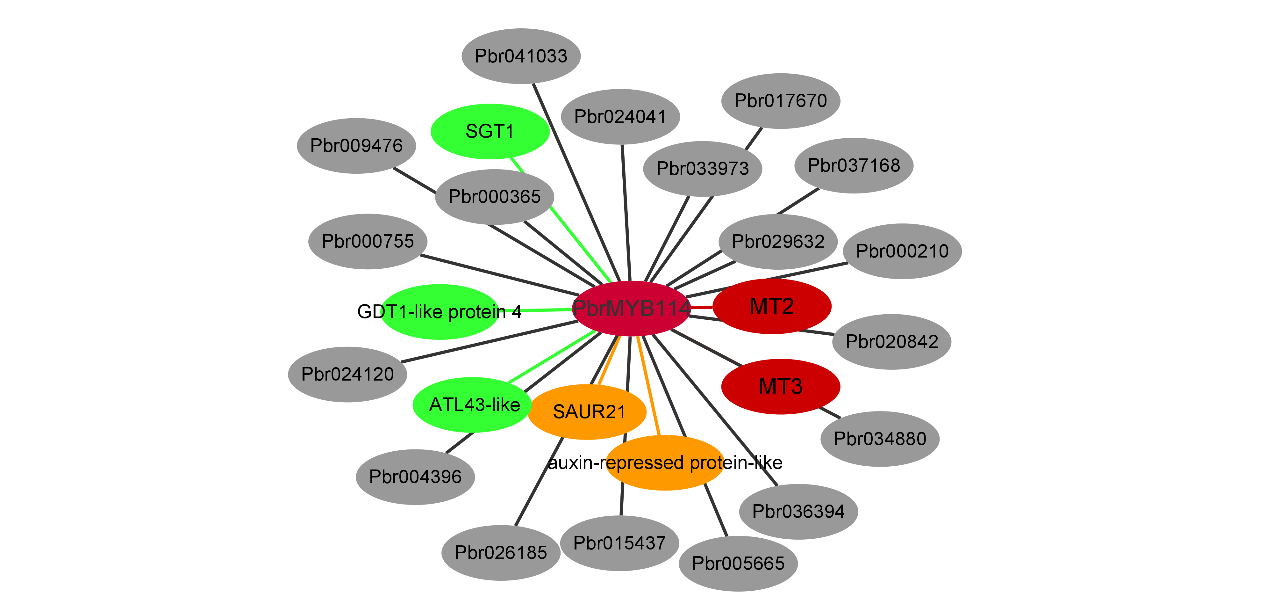


**Table S1. The list of primers used in this study**

| Primer name | Primer sequence (5'-3') |
| --- | --- |
| For Y2H assay | |
| T7-F | TAATACGACTCACTATAGGGGGCGA |
| AD-R | GCACGATGCAGTTGAAG |
| For gene cloning | |
| PbrMYB114-F | ATGAGGAAGGGTGCCTGG |
| PbrMYB114-R | TTAGTGACCACAGGTGCA |
| PbrMT2-F | ATGGCTCCTGATCTGAGCTAC |
| PbrMT2-R | TCACTTGCATTGCAGGGGTTG |
| PbrMT3-F | ATGTTGACACGAGAGGAG |
| PbrMT3-R | TTAGTGACCACAGGTGCA |
| For qRT-PCR | |
| PbrMYB114-F | GCCACATCCGTCATAAGACCTC |
| PbrMYB114-R | GCCACTCATGTGTAACCCTTC |
| PbrbHLH3-F | TTGTGGAGGGAAGTGGCGGT |
| PbrbHLH3-R | AGCTCCCTAAGTGTTTGCATCAC |
| Actin-F | CCATCCAGGCTGTTCTCTC |
| Actin-R | GCAAGGTCCAGACGAAGG |

**Amino Acid Sequences of targeted Proteins**

>PyMYB114（XP_018504231.2）

MWRAVMHVGGLMISNLVYIYHMEDSNLLGMMRKGAWTQQEDDILRQYVEKHGDGKWHQVPRETGLNRCRKSCRQRWLNYLKPNLKSGDFTEDEIDLIHRLQKLLGNRWSIIAGRLPGRTAGKVKNYWNSKQRKELEYMKDKSKERTKATSVIRPQPRRARVAIFQSEENCSRLLQTPSPPTENAIDSWKAMLHDTDNVDGTPFSSLGLGEDLFTNFWVEDIAQSTMVGMNSADEGLHMSGNFSFRENLWNLEEERTKI

>PbrMYB114

MRKGAWTQQEDDILRQYVEKHGDGKWHQVPRETGLNRCRKSCRQRWLNYLKPNLKSGDFTEDEIDLIHRLQKLLGNRWSIIAGRLPGRTAGKVKNYWNSKQRKELEYMKDKSKERTKATSVIRPQPRRARVAIFQSEENCSRLLQTSSPPTENAIDSWKAMLHDTDNVDGTPFSSLGLGEDLFTNFWVEDIAQSTMVGMNSADEGLHMSGNFSFRENFWNLEEEITKI

>*Pyrus pyrifolia* MYB10b（ALN66630.1）

MEDSNLLGIMRKGAWTQQEDDILRQCVEKHGDGKWHQVPRETGLNRCRKSCRQRWLNYLKPNLKSGDFTEDEIDLVHRLQKLLGNRWSIIAGRLPGRTAGKVKNYWNSKQRKELEYMKDKSKERTKATSVIRPQPRRARVAIFQSEENRSRLLQTSSPPTESAIDSWKTMLHDTDNVDGTPFSSLGLGEDLFTNFWVEDIAQSTMVGMNSADEGLHMSGNFSFRENLWNLEEEITKI

>*Malus domestica* MYB114-like（XP_028958896.1）

MISNLLGIMRKGAWTQQEDDILRQYVEKHGDGKWHQVPRETGLNRCRKSCRLRWLNYLKPNLKSGDFTEDEIDLIHRLQKLLGNRWSIIAGRLPGRTAGKVKNYWNSKQRKELEYMKDKSKERTKVTSVIRPQPRRARVAVLKSEENCSRLLQTSSPPTENAIDSWKTMLHDTDNIDGTPFTSLGLGEDLFTNFWVEDIAQSTTVGMNSADEGLHMSGNFSFRENLWNLEEERTKI

>*Arabidopsis thaliana* MYB113（NP_176811.1）

MGESPKGLRKGTWTAEEDILLRQCIDKYGEGKWHRVPLRTGLNRCRKSCRLRWLNYLKPSIKRGKLCSDEVDLVLRLHKLLGNRWSLIAGRLPGRTANDVKNYWNTHLSKKHDERCCKTKMINKNITSHPTSSAQKIDVLKPRPRSFSDKNSCNDVNILPKVDVVPLHLGLNNNYVCESSITCNKDEQKDKLININLLDGDNMWWESLLEADVLGPEATETAKGVTLPLDFEQIWARFDEETLELN

>*Pyrus communis* MYB10 （ABX71487.1）

MEGYNVNLSVRKGAWTREEDNLLRQCIEIHGEGKWNQVSYKAGLNRCRKSCRQRWLNYLKPNIKRGDFKEDEVDLILRLHRLLGNRWSLIARRLPGRTANDVKNYWYTRLRIDSRMKTVKNKSQETRKTNVIRPQPQKFIKSSYYLSSKEPILEHIQSAEDLSTPSQTSSSTKNGNDWWETLFEGEDTFERAACPSIELEEELFTSFWFDDRLSARSCANFPEEGQSRSEFSFSMDLWNHSKEE

>*Vitis vinifera* MYB (NP_001268002.1）

MEGSLGLRKGAWTSEEDHLLRKCVEKYGEGKWHQVPFRAGLNRCRKSCRLRWLNYLKPDIKRGKFTADEVDLMMRLHKLLGNRWALIAGRLPGRTSNDVKNYWNTHLRKKMVKDEVEETVKINAIRPRPRTFTKNLYWLERKELLENDQSELHIPRKPFSTTPPSEDGLNSWWESLFSDKEENKEITCSIERSANESISCLWDEQIAAMPEVGKTSITNGQIEWTDCSFDMDHLWDLINA

>*Prunus persica* MYB113 (XP_020420994.1)

MEGNNLLRVRKGAWTREEDELLRQYIQQYGEGKWHQVSLKAGLNRCRKSCRLRWLNYLRPNIKRGDFTEDEVDLMVRLRKLLGNRWSLIAGRIPGRTSNDVKNYWSTRLRKNKSSEAEKDKTLETTKTVILRPQPRTFSKKSNCLSSPAPTLQHIQLQENFNWPLPSSPPIENGIDEWKSQLADTNSVERAMCSGFQLEEDFFTNFWVENIAQNTGTGVNSADEGLLSYSDFSFHLWNFSKEK

>*Fragaria x ananassa* MYB10-like (WLF77577.1)

MEVRKGSWTKEEDHLLRNYIEKHGEGRWHKVPLQAGLNRCRKSCRIRWLNYLKPNIKRGDFEEDEVDLMIRLRKLLGNRWSLIAGRLPGRTSNDVKNYWSARRRKNINFGITNHNYKPPEVTKTTVIRPRPRTFTKSLHYLNAKVTTSNPIELGNSSSSTSPPIENGIDDWKTLLHEDVLTNFWVEDTASMSSIGVNSTEQGFEMDLWQFLQEETRQ

>*Solanum lycopersicum* ANT1(WDP81125.1 )

MNSTSMSSLGVRKGSWTDEEDFLLRKCINKYGEGKWHLVPMRAGLNRCRKSCRLRWLNYLRPHIKRGDFEQDEVDLILRLHKLLGNRWSLIAGRLPGRTANDIKNYWNTNLLRKLNTSKIVPREKINNKCGEISTKIEIIKPQPRKYFSSTKMNIVIFDEEEHCKEIISEKQTPDASMDNVDQWWTNLLENCNDDVEEDEEVVINYEKTLTSLLHEEISPPLNGEGNSIMQQGQTSHDSWGDFSLNLPPMQQGVQNDDWDDFSAEIDLWNLLD

>*Diospyros kaki* MYB (BAI49718.1 )

MRKPCCDKQDVNKGAWSRQEDQKLIDYIEKHGEGNWRLLPKAAGLVRCGKSCRLRWINYLRPDLKRGNFGEDEEDLIVKLHALLGNRWSLIAGRLPGRTDNEVKNYWNSHLRKKLMSKGIDPDKHRLRQSTVVHLQPVVSHERGDKFSDAATSAHDNHKPISELPDVNLNLSLSIN

>*Ziziphus jujuba* MYB1 (XP_048332153.2 )

MIISINMLRRQYTMDGFLGLRKGTWTKEEDDLLKQCVEKYGEGKWHQVPRRAGLNRCRKSCRLRWLNYLKPDIKRGEFTMDEVDLLLRLHKLLGNRWSLIAGRLPGRTANDVKNYWNTHLQKKFLPNYEKNKNKEINADKKTHGCDTKVIKPRPWTFAKRMSENYASTIIMDNVNRPHQQQNFHQKSLEEDLDWWENLLHNTTPNKGATYNVSGSEGDAISKLPDEDQEGRELVQPSNIIGDVLVEDNQSFWRDFNIDMDLWDL
