# Supplementary material for: Screening and verification of proteins that interact with the anthocyanin-related transcription factor PbrMYB114 in ‘Yuluxiang’ pear
Source: PeerJ. 2024 Jun 14;12:e17540. doi: 10.7717/peerj.17540 (PMC11182023; doi:10.7717/peerj.17540)

Figer 1A The PbrMYB114 cDNA fragment

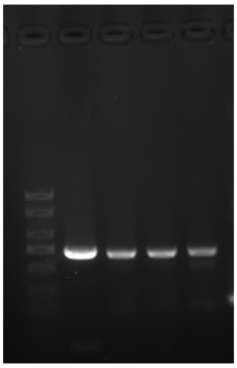

Figer 2A Evaluation of the integrity of total RNA from 'Yuluxiang' pears;

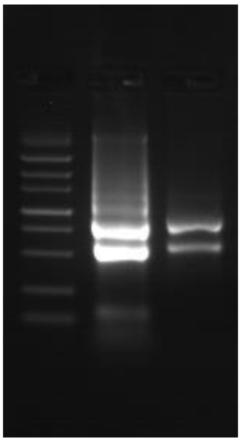

Figer 2B Double-stranded cDNA was evaluated by 1% agarose gel electrophoresis following normalization.

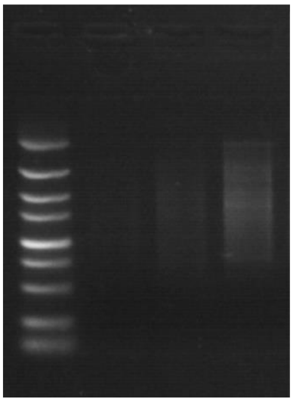

Figer 2D PCR-based assessment of insertion fragments

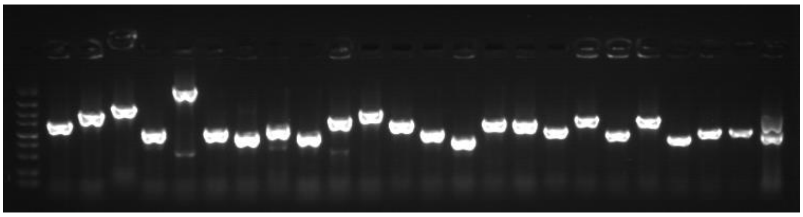

Supplement: Supplemental Information 2 [file peerj-12-17540-s002.pdf]
